# Cognition-emotion interactions: patterns of change and implications for math problem solving

**DOI:** 10.3389/fpsyg.2014.00840

**Published:** 2014-07-31

**Authors:** Kelly Trezise, Robert A. Reeve

**Affiliations:** Melbourne School of Psychological Sciences, University of MelbourneParkville, VIC, Australia

**Keywords:** working memory, worry/anxiety, math problem solving, individual differences, change

## Abstract

Surprisingly little is known about whether relationships between cognitive and emotional states remain stable or change over time, or how different patterns of stability and/or change in the relationships affect problem solving abilities. Nevertheless, cross-sectional studies show that anxiety/worry may reduce working memory (WM) resources, and the ability to minimize the effects anxiety/worry is higher in individuals with greater WM capacity. To investigate the patterns of stability and/or change in cognition-emotion relations over time and their implications for problem solving, 126 14-year-olds’ algebraic WM and worry levels were assessed twice in a single day before completing an algebraic math problem solving test. We used latent transition analysis to identify stability/change in cognition-emotion relations, which yielded a six subgroup solution. Subgroups varied in WM capacity, worry, and stability/change relationships. Among the subgroups, we identified a high WM/low worry subgroup that remained stable over time and a high WM/high worry, and a moderate WM/low worry subgroup that changed to low WM subgroups over time. Patterns of stability/change in subgroup membership predicted algebraic test results. The stable high WM/low worry subgroup performed best and the low WM capacity-high worry “unstable across time” subgroup performed worst. The findings highlight the importance of assessing variations in cognition-emotion relationships over time (rather than assessing cognition or emotion states alone) to account for differences in problem solving abilities.

## INTRODUCTION

The impact of cognitive states on emotion and of emotional states on cognition has most often been examined in cross-sectional studies. Research shows that individuals with limited working memory (WM) capacity may experience difficulty regulating anxiety levels (Hofmann et al., 2011), and anxiety/worry may reduce WM resources (Eysenck et al., 2007). Surprisingly little is known about the stability/change in cognitive and emotional states over time, or how different kinds of change relationships affect problem solving abilities. Moreover, it appears that the pattern of stability/change in emotion-cognition relations differs across individuals over time (Pnevmatikos and Trikkaliotis, 2013). We suggest that a better understanding of the nature of these different patterns would provide a more complete characterization of the impact of changes in emotion-cognition on problem solving. To this end, we employ a latent transition model to characterize patterns of stability/change in WM-worry and to determine their impact on math problem solving.

Theories of emotion regulation propose that WM plays an important role in the regulation of emotions (Van Dillen and Koole, 2007; Hofmann et al., 2011, 2012). Individual differences in WM capacity are linked to the ability to regulate emotional responses (Schmeichel et al., 2008; Schmeichel and Demaree, 2010). Schmeichel et al. (2008), for example, found that when instructed to neutralize an emotional response while viewing an emotive film, individuals with a greater WM capacity showed less emotional facial expressions and reported more neutral mood, compared to those with a smaller WM capacity. In another study, individuals with low WM showed an increase in negative affect in response to negative feedback, whereas high WM individuals did not show an increase (Schmeichel and Demaree, 2010). These findings suggest that cognitive level affects change in emotion; specifically, individuals who have greater WM capacity are better able to control their emotional states. Moreover, it has been suggested that temporary reductions in cognitive abilities (such as WM) lead to poorer regulation of emotions (e.g., more susceptible to increases in negative emotions such as worry – Hofmann et al., 2012).

Emotion states may also affect cognitive abilities (Richards and Gross, 2000; Eysenck et al., 2007). According to the processing efficiency theory and attentional control theory (ACT), worry (the cognitive component of anxiety) is thought to require processing capacity, thus reducing WM capacity available for other tasks (Eysenck and Calvo, 1992; Ashcraft and Kirk, 2001; Derakshan and Eysenck, 2009; Eysenck and Derakshan, 2011). Schoofs et al. (2009), for example, found that individuals exposed to a cold-induced stress manipulation, showed an immediate decrease in their WM, whereas individuals in a neutral condition showed no change to WM. Pnevmatikos and Trikkaliotis (2013) examined the impact of induced emotions on the ability to inhibit incorrect responses in an arrow direction judging task. 8- to 12-year-olds completed the task under neutral and induced emotion conditions twice, 3 weeks apart. In the neutral condition, children completed the same arrow judgment task on both occasions. In the induced emotion condition, task instructions were designed to induce frustration, following which the arrow judging task was completed. Later, pleasant experiences were presented to induce a positive mood state, and the arrow judging task completed again. Pnevmatikos and Trikkaliotis (2013) findings show the pattern of judgment errors were associated with emotion states for most, but not all children. A question of some interest is why do some individuals remain impervious to emotional manipulations? What factors allow some individuals better able to control the impact of emotions than others?

The interaction between cognition and emotion likely depends on cognitive demands. Blair et al. (2007), for example, found that individuals were slower to detect which of two arrays displayed the greater number of digits, when a picture presented before the to-be-judged arrays contained an emotional (compared to a neutral) picture. The difference between emotional and neutral trials was greatest when the to-be-judged arrays were incongruent, compared to when they were congruent. In other words, the emotion manipulation affected judgment abilities, and the effect was greater when cognitive demand increased. The findings from Blair et al. (2007) show that emotion and cognition interact to impact the how information is processed.

Cognition-emotion relationships have often been examined in the context of math. For example, to investigate the effects of WM on emotion regulation, arithmetic tasks are used to manipulate WM demands (Van Dillen and Koole, 2009; Van Dillen et al., 2009; Kanske et al., 2011), and math-specific anxiety has been associated with reduced WM capacity and slow and inaccurate arithmetic problem solving (Ashcraft and Faust, 1994; Faust et al., 1996; Ashcraft and Kirk, 2001; Mattarella-Micke et al., 2011; Suárez-Pellicioni et al., 2013). Interactions between anxiety and WM capacity have been shown to affect math problem solving and reasoning abilities (Owens et al., 2014). Owens et al., for example, found that for individuals with relatively small digit and spatial spans, high anxiety negatively affected math reasoning; however, high anxiety, with high WM span, positively affected reasoning ability.

Math problem solving places demands on WM (Raghubar et al., 2010), and elicits anxiety in some individuals (Ashcraft and Faust, 1994). However, WM-anxiety relationships are most commonly examined in the context of arithmetic. Arithmetic is introduced prior to school, and by adolescence is highly familiar and well-practized. Hence, examining cognition-emotion interactions with arithmetic may underestimate possible effects of WM and/or anxiety, compared to more advanced math, such as algebra. Algebra is introduced during early adolescence years. Many students find the learning of algebraic principles particularly troublesome (Knuth et al., 2005). Algebra is known to cause anxiety in students (Uusimaki and Nason, 2004; Ng, 2012). Algebra is hypothesized to be WM demanding as it requires maintenance of math expressions, retrieval of algorithms and math facts, and inhibition of arithmetic responses (Tolar et al., 2009).

Identifiably different patterns of emotion-cognition interactions in an algebraic context have been found in at least one study. Trezise and Reeve (2014) used latent profile analysis to identify different WM-worry subgroups at a single time point. Worry was assessed as students made algebraic judgments, and WM was assessed with an alphanumeric dual span task. Trezise and Reeve (2014) identified four WM-worry subgroups: high WM with low worry, moderate WM with low worry, moderate WM with high worry, and low WM with high worry, each of which were related to algebraic problem solving in predictable ways. WM/anxiety relationships in math problem solving are typically examined assuming trait properties, but given that cognition and emotion affect each other, a question of some interest is whether patterns of cognition-emotion interaction remain stable or differ across time?

The present study is designed to determine whether different patterns of cognition-emotion relationships remain stable or change over a short period of time, and whether these patterns are associated with differences in algebraic problem solving. Previous research has demonstrated cognition and emotion interact: individuals with high WM show less emotional variation than and low WM individuals (Schmeichel and Demaree, 2010), and individuals show lower WM after an emotion or stress induction (Schoofs et al., 2009). Little research has investigated the possibility that the nature of these interactions change over time. Of interest is whether particular cognition-emotion relationships are more/less stable than others and, in particular, whether initial differences in the cognition-emotional relationship differ over time. If some individuals, because of their initial WM or worry, are more likely to remain stable over time and others more likely to change, it suggests that differences between individuals may increase over time. Furthermore, if individuals with high WM and low worry are more resistant to changes, and individuals with low WM and/or high worry are more vulnerable to changes, it suggests that the disadvantage of low WM and/or high worry individuals may amplify over time. To investigate these issues, we use similar stimuli to those used by Trezise and Reeve (2014) to examine high school students’ algebraic WM and algebraic worry twice in a single day as they studied for an end-of-the day algebra test.

We use latent transition analysis (LTA) to examine patterns of change/ stability. LTA is a person-centered approach for identifying clusters of individuals who share similar response patterns (Nylund, 2007; Bray et al., 2010; Lanza et al., 2010). More specifically, LTA models subgroup membership over discrete time intervals, and does not rely on common modeling assumptions (e.g., the presence of normal distribution and linear relationships). Moreover, it does not involve a predetermined number of subgroups, rather, it assumes there are pre-existing natural subgroups (i.e., a discrete latent variable) and the aim of analysis is to identify them (i.e., the number and characteristics of the unobserved latent variable). To our knowledge, LTA has not been used to examine stability/change relationships between cognition and emotion in math; however, it has been used to examine stability and change in personality development (Meeus et al., 2011), academic motivation (Marcoulides et al., 2008), and Piagetian stages of cognitive development (Dolan et al., 2004). Meeus et al. (2011), for example, used LTA to characterize transitions in personality in adolescence: they identified personality types associated with change and others that marked “the end point” of personality development.

We aim to use LTA to provide an *integrative model* of WM-worry change relationships, and their implications for problem solving. We do this is several stages. First, we find the best fitting model of WM-worry relationships over time. Second, we characterize students’ initial WM-worry pattern, and third, we characterize students’ stability/change in their WM-worry relationships over time. Finally, the end point of this change (i.e., final WM-worry pattern) is then regressed on problem solving ability.

Our research addresses three questions. First, can we identify WM-worry relationships similar to those identified by Trezise and Reeve (2014)? On the basis of Trezise and Reeve’s (2014) research, we expect to identify four WM-worry subgroups, including a high WM/low worry subgroup, moderate WM/low worry, moderate WM/high worry, and low WM/high worry.

Second, does WM-worry subgroup membership change over time? We use a LTA model (Vermunt and Magidson, 2013a) to identify WM-worry subgroups and the stability/change of subgroup membership over the two test occasions. In particular, we asked the question: if an individual exhibits a particular WM-worry relationship at Time 1, what is the probability that they will exhibit the same or a different WM-worry relationship at Time 2? More specifically, we expect that higher levels of worry will be associated with declines in WM, and that low WM would be associated with increases in worry. Specifically, we expect that high WM and low worry subgroups will be more likely remain stable over time and individuals initially in lower WM and higher worry subgroups more likely to change subgroups over time.

Third, we expect that WM-worry relationships would predict problem solving ability. Individuals with high WM capacity or low math anxiety show faster and more accurate arithmetic problem solving ability (Ashcraft and Faust, 1994; Faust et al., 1996; Imbo and Vandierendonck, 2007; Imbo and LeFevre, 2010; Alloway and Passolunghi, 2011; Caviola et al., 2012; Geary et al., 2012; Simmons et al., 2012). Given that both WM and worry/anxiety affect math problem solving, if worry and/or WM change over time, a question of some interest is whether changes to worry and/or WM affect math problem solving abilities. We expected that high WM and low Worry would be associated with accurate and fast algebraic problem solving, and that low WM and high Worry would be associated with inaccurate and slow algebraic problem solving.

## MATERIALS AND METHODS

### PARTICIPANTS

One-hundred-twenty-six 14-year-olds (*M* = 14 years, 4 months, SD = 4 months; 89 boys, 37 girls) attending mixed gender schools in an Australian city, participated. Common to Australian urban high schools, the sample comprised students from diverse multicultural and socio-economic backgrounds. According to school personnel, none of the participating students had identified learning difficulties, and all had normal or corrected to normal vision. The research was approved by, and conducted in accordance with the requirement of, the authors’ University’s Human Research Ethics Committee. Approximately 95% of students invited to participate in the research did so.

### PROCEDURE

**FIGURE 1:**
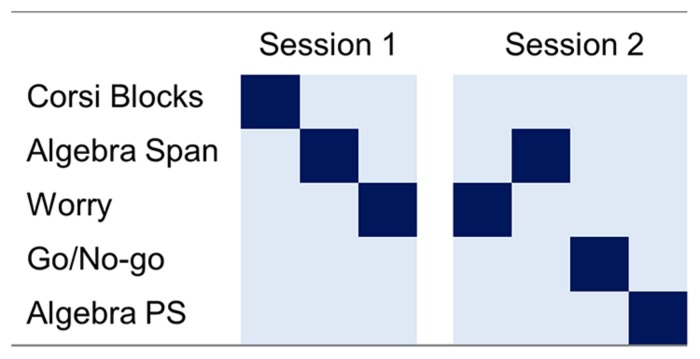
**Study design and task sequence for testing sessions one and two**. Dark squares indicate task completed. Task sequence is left to right. The figure is schematic, and not representative of time intervals; session one occurred in the morning, and session two in the afternoon.

Students completed three algebraic tasks: an (1) algebraic WM, (2) algebraic judgment/worry (worry); and (3) algebraic problem solving test; and two domain general tasks: (1) Corsi Block (visuo-spatial working memory, VSWM), and (2) Go No-Go (response inhibition). Tasks were completed in two sessions in a single day (see **Figure 1** for test sequences). In Session 1, students completed tasks in a fixed order: (1) Corsi Block, (2) WM, and (3) worry. In Session 2 the order was: (1) worry, (2) WM, (3) Go/No-Go, and (4) problem solving. As WM is thought to directly impact math reasoning, we deemed it important to establish WM abilities at the beginning of testing, and immediately prior to the problem solving task. All tasks were completed on 15” laptop computers running Inquisit Web 3.0.6.0 software (2011).

### MATERIALS

**FIGURE 2:**
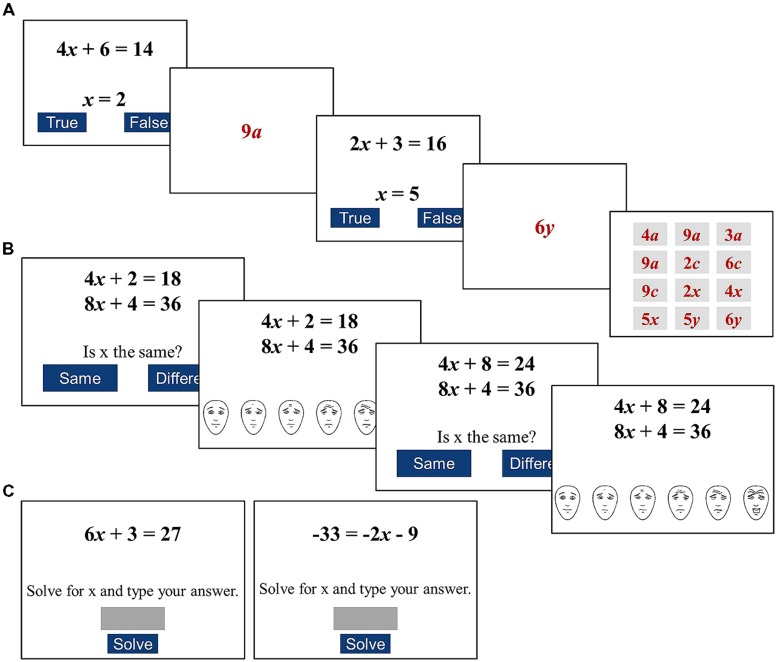
**Example stimuli for the (A) WM, (B) worry, and (C) problem solving tasks**. A two trial sequence on the algebraic WM task is depicted: two trials each containing a processing (correct/incorrect) and memory (algebraic term) phase, and memory terms on a recognition screen. Algebraic worry task involves a judgment about the equivalence of two equations, followed by a worry rating; easy (left) and difficult (right) trials are shown. Easy, and hard (left to right) problems for the algebraic problem solving task are shown.

The *Algebraic WM* task was based on Turner and Engle’s (1989) operation span task, modified to use alphanumeric symbols. The task was designed to examine domain-relevant WM by assessing the ability to both appraise algebraic statements, and remember alphanumeric symbols (see **Figure 2**). Each trial consisted of an algebraic statement appraisal, and the presentation of an algebraic symbol to remember. Algebraic statement appraisal required students to judge the accuracy of an algebraic statement (e.g., 3*y* + 2 = 20; *y* = 2) and indicate their judgment by pressing a key on their computer (students were given 15 s to respond). They were instructed to make judgments of the accuracy of algebraic statements, rather than solve equations. An alphanumeric symbol then appeared on the screen (e.g., “4*x*”) for 1600 ms. After *n*-trials, a 3 × 4 matrix consisting of 12 alphanumeric symbols appeared onscreen (see **Figure 2**). Students were instructed to select symbols in the matrix that had been presented in the trials, in the order than were presented.

Students received instructions and training for the appraisal and span components separately following which they completed two practice sets, prior to Session 1. Students completed two sequences of two-, three-, four-, and five-trial sets (i.e., 2 × 2, 3, 4, and 5 trial sets; see **Figure 2**). Order of presentation of set length was randomized. While students were instructed to do their best on both the appraisal and span components of the task, we were interested in the proportion of alphanumeric symbols correctly recognized. We used the partial scoring procedure suggested by Conway et al. (2005) and Redick et al. (2012) which has good psychometric properties (e.g., test–retest and internal consistency).

The *Algebraic Worry* task was used to assess worry students experienced while making algebraic judgments. Students were shown pairs of algebra equations of the form m*x* + c_1_ = c_2_ and judged whether the value of the variable (*x*) in the two equations had the same value (equivalent equations), or different values (non-equivalent equations; see **Figure 2**). The design of the judgment procedure was based on Canobi et al. (1998, 2003) arithmetic equation judgment task. The task examines students’ ability to notice the presence of commuted relationships in two equations. Students were instructed to compare between the two equations, rather than solve the equations. Following each judgment, the Faces Anxiety Scale (Bieri et al., 1990) appeared on the screen and students rated their Worry experienced while making their judgment. The Faces Anxiety Scale comprised six faces depicting increasing Worry (neutral to extreme Worry) and students selected the face that corresponded to their level of worry.

The initial session included a familiarization section, in which students underwent training for the equivalence judgments and how to use the worry scale. Students also received training on how to use the worry scale: specifically, they were given non-math and math examples as practice. Training was not given in the second session, because we assumed familiarity carried over from the first session. Students were not informed of their judgment accuracy. In Session 1, 16 equations pairs were rated and in Session 2, 20 pairs were rated.

In the *Algebra problem solving* task students solved linear algebra equations (see **Figure 2**). The task comprised eight easy and eight hard equations presented in random order. The aim of the problem solving task was to assess students’ ability to solve algebraic equations. The structure of the equations was based on the format that students encounter in their math classes. Difficulty was varied using known properties of equivalence relationships (see Humberstone and Reeve, 2008; Trezise and Reeve, 2014). Easy equations required a less sophisticated understanding of the equivalence sign, and comprised three-terms problems with a variable, a coefficient and a constant on the left side of the equivalence sign, and a constant on the right (e.g., m*x* + c_1_ = c_2_). Hard equations required a more sophisticated understanding of the equivalence sign, and comprised negative values, and variables (with coefficient) on the left or right side of the equivalence sign (see Alibali et al., 2007; Humberstone and Reeve, 2008). Each hard equation also included negative integers. Solutions ranged between -9 and +12. Students were given up to 15 s to respond. Correctness and median response times (RTs) were recorded.

The Corsi-Blocks task (Milner, 1971) is a measure of visuo-spatial WM often used in math cognition research (e.g., Andersson and Lyxell, 2007; Bull et al., 2008; Krinzinger et al., 2012). A digital version of the task was used. Nine cubes were arranged irregularly on the screen. Each trial consists of a sequence of blocks that light up one block per second. Once a sequence had finished, participants reproduce the sequence, by clicking those blocks in the same order. Two trials of each sequence length were shown, and sequences gradually increased in length from two to nine blocks. If participants correctly reproduced at least one of the trials of the same sequence length, then the sequence length was increased by one. The task continued until both trials of the same sequence-length were incorrect, or participants completed trials with the largest sequence. We recorded the number of the longest sequence remembered (2–9).

The *Go/No-Go* task is a widely used measure of inhibition (Blaye and Chevalier, 2011). In the Go/No-Go task a black orienting cross appear in the center of the screen, followed by either a red or a blue dot (500, 1000, or 1500 ms after the black cross). Participants pressed a computer key as fast as possible only if a red dot appeared. The task comprised 36 trials, with half No-Go trials. We were interested in the proportion of correctly withheld responses to assess inhibition, and RTs for correct Go responses to assess processing speed.

### DATA ANALYSIS

**FIGURE 3:**
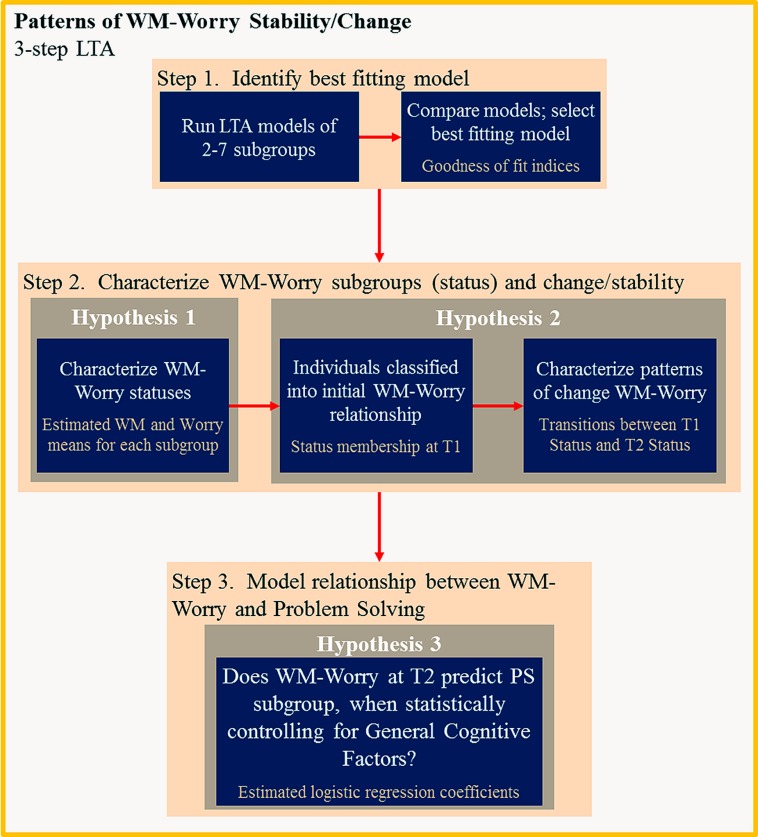
**Flow chart of data analysis process**. T1, Time 1; T2, Time 2; PS, problem solving.

Data were analyzed using a three-step LTA approach (see **Figure 3**; Vermunt and Magidson, 2013b). First, we sought to identify the latent transition model of WM-worry relationships over time. We then sought to characterize stability/change subgroups using the LTA model. Finally, we investigated the association between WM-worry LTA subgroups and algebraic problem solving ability.

Latent transition analysis identifies subgroups of individuals based on their response patterns on several variables, and changes in subgroup membership over time. It is a longitudinal extension of latent class analysis: density-based classification is used to identify of subgroups of individuals who share similar response patterns. LTA involves running models of increasing numbers of subgroups. Models are compared on “combination of statistical criteria, parsimony, and interpretability” (Collins and Lanza, 2010, p. 82) to identify the model that best fits the data. In other words, the model (and number of subgroups) is determined post-analysis, through examination of goodness of fit indices.

Goodness of fit statistics includes the log-likelihood, Akaike information criteria with correction for finite sample sizes (AICc), Bayesian information criteria (BIC), and sample-size adjusted BIC (aBIC). The log-likelihood reflects absolute fit of how well the model fits the observed data (e.g., WM and worry). The AICc, BIC, and aBIC represent the balance between fit and parsimony; they are based on the log likelihood estimates, but also contain a penalty component that increases with the number of estimated parameters purely serving to improve model fit, to discourage over-fitting of a model (Vermunt and Magidson, 2013b). The AICc, BIC, and aBIC values are compared between models, where lower values indicate optimal balance. Entropy, a measure of how well the model classifies individuals into subgroups, is also estimated. The entropy value describes how well the classes are separated and is useful for judging the confidence in the model of assigning individuals to clusters (Celeux and Soromenho, 1996; Lanza and Collins, 2007). If the clusters are more distinct, entropy tends to be closer to one (Celeux and Soromenho, 1996; Lanza and Collins, 2007); values greater than eight are considered good classification (Clark and Muthén, 2009).

In LTA, subgroups are referred to as latent statuses. LTA estimates three types of parameters. Conditional (response) density parameters are estimates of response patterns for each observed variable, conditional on membership of a particular latent status. In other words, conditional density parameters are used to characterize statuses. Initial-state probabilities are the probability of each individual belonging to each latent status at the initial time point (Vermunt and Magidson, 2013a). In the current research context, an individual with high WM and low worry responses is likely to belong to the high WM and low worry status. Individuals may change status membership over time. In other words, an individual with a particular WM-worry relationship on the first test occasion may have that same WM-worry relationship on the second occasion and stay in the same status, or they may show a different WM-worry relationship and move to a different status. Latent transition probabilities are estimated for each individual to determine the probability of being in a particular latent status at one time, conditional on their status membership at the previous time point (Vermunt and Magidson, 2013a). Individuals are classified as belonging to the status at Time 1 for which the latent status probability is highest, and to the Time 2 latent status for which the latent transition probability is the highest. In sum, the LTA calculates initial status probabilities to classify individuals to a status at the initial time point, and transition probabilities between Time 1 and Time 2, to classify individuals to a status at Time 2. LTA also produces conditional density parameters which are used to characterize the each status by the response patterns for each observed variable.

The third step in LTA examines the association between the assigned class memberships and external variables. This step is traditionally completed using multinomial regression or ANOVA, once individuals have been assigned to latent subgroups. Classification errors are introduced when individuals are assigned to latent statuses, which results in underestimation of the relationship between latent statuses and external variables (see Bolck et al., 2004). To overcome these issues, adjustments to the three-step procedure are made by correcting classification errors and estimating a logistic regression model between the latent statuses and external variables with reweighted frequencies (see Bolck et al., 2004; Vermunt, 2010; Bakk et al., 2013).

In the present study, for the first and second steps of the LTA, eight observed variables were included in the analysis: proportion of algebraic terms correctly recognized for small (2- and 3-symbol) and large (4- and 5-symbol) sets, and easy and hard worry ratings from time 1 and time 2. Latent statuses are expected to represent different WM-worry relationships at a single assessment. We estimated six models (2 to 7 statuses) which were compared using goodness-of-fit statistics to identify the best-fitting model. We then examined the conditional response density parameters to characterize WM-worry statuses, and the initial state and transition probabilities to characterize WM-worry relationships and changes over time.

To examine the relationship between WM-worry status and algebraic problem solving abilities, we used a three-step procedure in Latent GOLD, in which WM-worry status membership predicts problem solving abilities, with general cognitive factors as covariates. We used proportional assignment, which treats individuals as belonging to each of the classes with weights equal to the posterior membership probabilities (instead of assigning them to a single status for which their posterior membership probability is highest, see Vermunt, 2010), and a BCH adjustment (Vermunt and Magidson, 2013a; Bakk and Vermunt, under revision).

## RESULTS

Descriptive statistics, and correlations for WM and worry tasks, problem solving, and general cognitive factors are reported in **Tables 1 and 2**, respectively. There are four sections to the results. First, the model that best fits patterns of WM-worry relationships is identified. Second, subgroups (i.e., statuses) that characterize different WM-worry relationships are described. Third, patterns of change in WM-worry relationships over a short period of time are described. Finally, we examine how these WM-worry relationships predict algebraic problem solving abilities. To assist comprehension of the findings, each WM-worry status is represented by the same color in all figures.

**Table 1:** Means and standard deviations for working memory, worry/judgment, algebra problem solving performance, VSWM, inhibition, and basic response time.

		Time	*M*	SD	*M*	SD	*M*	SD
			**Small sets**		**Large sets**		**Total**	
Algebra Span	Recall	1	0.700	0.287	0.410	0.242	0.513	0.236
		2	0.535	0.394	0.335	0.279	0.406	0.305

			**Easy**		**Hard**		**Mean**	
Judgment/Worry	Worry	1	1.487	1.196	1.677	1.149	1.583	1.148
		2	1.520	1.250	1.532	1.239	1.526	1.228
	Accuracy	1	0.717	0.240	0.672	0.254	0.696	0.198
		2	0.666	0.215	0.656	0.239	0.662	0.203

			**Accuracy**		RT^*^			
Algebraic PS	Easy	2	0.461	0.378	5.828	4.527		
	Hard	2	0.186	0.238	7.081	5.623		

			**Time 1**					
Corsi-Blocks	VSWM span	1	6.350	1.082				
Go/No-Go	Inhibition	2	0.863	0.210				
	Basic RT	2	345.822	72.510				

* Includes RT for both correct and incorrect responses.

**Table 2:** Correlation for algebraic WM recognition accuracy, worry ratings, VSWM, problem solving accuracy and response time, VSWM, inhibition and basic response time.

		Algebraic WM		Algebraic worry		VSWM	Problem solving		Inhibition	RT
		T1	T2	T1	T2		Accuracy	RT		
Algebraic WM	T1	-								
	T2	0.515^**^	-							
Algebraic worry	T1	-0.321^**^	-0.185^*^	-						
	T2	-0.325^**^	-0.290^**^	0.759^**^	-					
VSWM		0.033	0.117	-0.203^*^	-0.306^**^	-				
Problem solving	Accuracy	0.241^**^	0.521^**^	-0.312^**^	-0.371^**^	0.261^*^	-			
	RT	-0.068	0.274^**^	0.195^*^	0.039	0.044	0.396^**^	-		
Inhibition		0.309^**^	0.339^**^	-0.210^*^	-223^*^	0.038	0.331^**^	0.105^*^	-	
RT		0.091	0.164	-0.044	-0.022	-0.188	0.064	0.081	0.350^**^	-

* *p* < 0.05, **p < 0.001.

### CHARACTERIZATION OF PATTERNS OF WM-WORRY AND CHANGE/STABILITY

#### What is the best representation of patterns of WM-Worry relationships?

Small and large WM capacity and lower and higher worry ratings from Time 1 and Time 2 were entered into the LTA. Models comprising two to seven latent statuses were estimated and models compared to select the best fitting model. **Table 3** presents model fit information. The BIC and AICc were optimal for the 6-status model, representing a good balance between model fit and parsimony. Entropy for the all models was significant, indicating good classification of individuals into latent statuses. We also conducted a bootstrap log-likelihood test of model parsimony between the 5-status and 6-status models, which revealed a revealed a significant difference (-2LL = 89.624, Δ*df* = 15, *p*< 0.001), indicating that the 6-status model was a better fit of the data. We then compared the 5- and 6-status models in terms of conceptual interpretability and deemed the 6-status model to be show better interpretability. We therefore considered the 6-status model to be the best model, in terms of statistical fit, parsimony, and theoretical interpretability, of WM-worry relationships over time.

**Table 3:** Model fit information used in selecting the latent transition analysis model.

*N* status	*N*par	LL	BIC	aBIC	AICc	Entropy
2	15	54.870	-37.196	-84.6304	-105.376	0.963
3	24	161.680	-207.288	-283.185	-311.479	0.977
4	35	217.641	-266.012	-376.693	-407.282	0.964
5	48	279.352	-326.561	-478.353	-497.613	0.944
**6**	**63**	**324.164**	-**343.641**	-**542.867**	-**518.263**	**0.936**
7	80	356.532	-326.162	-579.146	-425.064	0.934

*N* status, number of latent statuses; *LL*, log likelihood; *Npar*, number of parameters in model; *BIC*, Bayesian information criterion, *aBIC*, adjusted BIC; *CAIC*, consistent Akiake information criterion. Bold indicates best fitting model.

#### Characterisation of WM-Worry relationships

**FIGURE 4:**
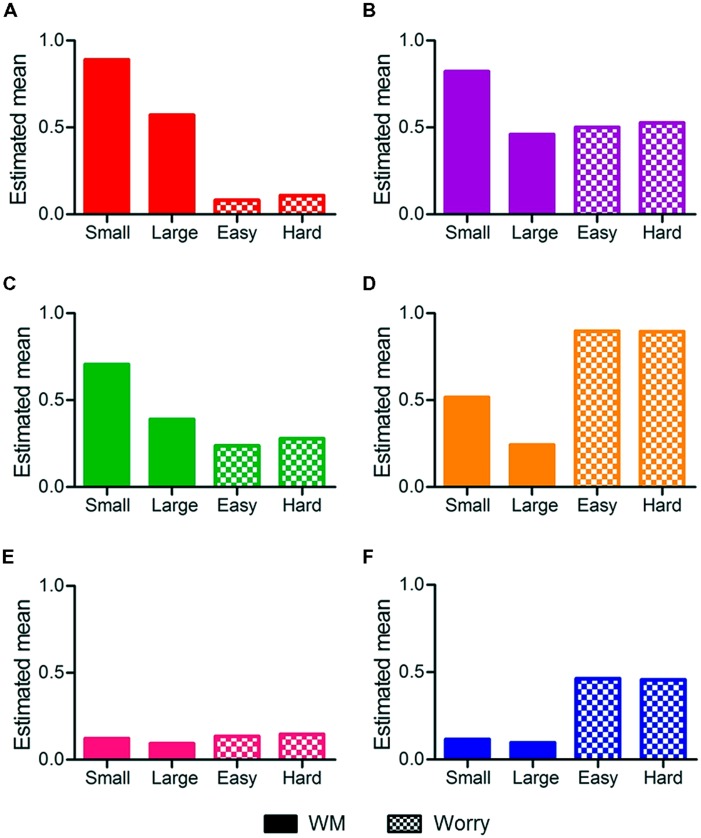
**Statuses from the LTA and mean scores on WM small (2 and 3), WM large (4 and 5) sets, and Worry for easy and hard judgments**. Statuses are **(A)** high WM/low worry, **(B)** high WM/high worry, **(C)** moderate WM/low worry, **(D)** moderate WM/high worry, **(E)** low WM/low worry, and **(F)** low WM/high worry.

Profiles for each status are presented in **Figure 4**. The statuses (and divisions between high/low, etc.) are not pre-determined, but an outcome of the modeling processes. Two statuses displayed high WM: one with low worry, labeled high WM/low worry, and one with high worry, labeled high WM/high worry. Two statuses were characterized by moderate WM performance: one with low worry (labeled moderate WM/low worry), and the second with very high worry (labeled moderate WM/high worry). There were two statuses with low WM: a low worry and a high worry status, labeled low WM/low worry and low WM/high worry, respectively.

#### Patterns of change predicted by initial WM-worry relationship

**FIGURE 5:**
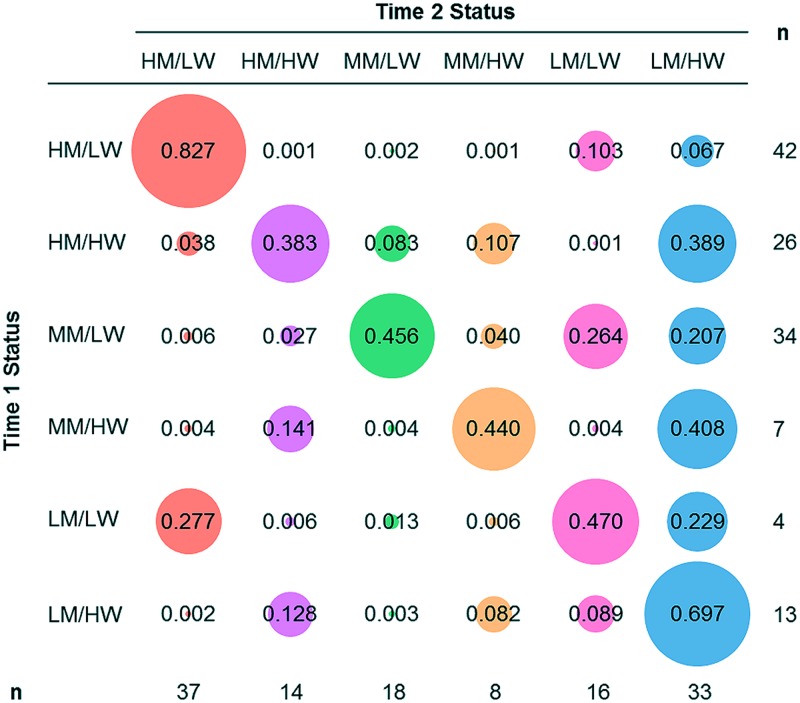
**Latent transition probabilities and number of students in each status at Time 1 and Time 2**. Probabilities indicate Time 2 status membership based on Time 1 status membership. Area of bubbles corresponds to probability size; bubble color represents Time 2 status. The left-to-right diagonal indicates the probability of individuals staying in the same status from Time 1 to Time 2. All other probabilities (i.e., not on the left-to-right diagonal) are indicate probabilities of individuals change statuses from Time 1 to Time 2. Statuses are HM/LW (high WM/low worry), HM/HW (high WM/high worry), MM/LW (moderate WM/low worry), MM/HW (moderate WM/high worry), LM/LW (low WM/low worry), and LM/HW (low WM/high worry).

Transition probabilities, and number of students identified for each status at Time 1 and Time 2 are shown in **Figure 5**. In **Figure 5**, each row represents a Time 1 status, and each column represents a Time 2 status. The matrix displays the transition probabilities of individuals in each Time 1 status belonging to each Time 2 status. The left-to-right diagonal indicates the probability of individuals staying in the same status from Time 1 to Time 2. All other probabilities (i.e., not on the left-to-right diagonal) indicate probabilities of individuals’ change statuses from Time 1 to Time 2. Bubble area corresponds to probability size and color corresponds to Time 2 status. The probabilities of each row sum to 1. Probabilities and bubble size represent the probability of an individual belonging to the (row) status at Time 1 is likely to be in the (column) status at Time 2.

The high WM/ low worry status had a large number of students at Time 1. Transition probabilities indicate a high proportion of those students remained in the high WM/low worry status at Time 2 and a small proportion moved into the low WM/low worry or low WM/high worry statuses. That is, individuals with high WM/ low worry displayed relatively stable WM-worry relationships. For students in the high WM/high worry status at Time 1, there was an equal probability for those students to remain in the high WM/high worry status or to move to the low WM/ high worry status at Time 2. For the moderate WM/low worry status, transition probabilities indicate that students remained in the moderate WM/low worry status between Time 1 and Time 2, or moved to low WM/low worry or low WM/high worry statuses at Time 2. Both the high WM/high worry and moderate WM/low worry statuses had a large number of individuals at Time 1, but status membership almost halved over time. In sum, a high WM with high worry, or a moderate WM with low worry relationship is unstable over time. The moderate WM/high worry status contained a small number at both Time 1 and Time 2: students either stayed, or transitioned to the low WM/high worry status at Time 2. The low WM/low worry status was small at Time 1 (*n* = 4), but many students moved into the status at Time 2. Membership to the low WM/high worry status increased over time: students belonging to the status at Time 1 were likely to stay, and students from other statuses at Time 1 were likely to transition into the status at Time 2.

Overall, the high WM/ low status was relatively stable; students with higher worry, or lower (i.e., moderate) WM were likely to transition to lower WM statuses at Time 2. Few students transitioned to a higher WM, or lower worry status.

### DOES WM-WORRY CHANGE/STABILITY PREDICT PROBLEM SOLVING?

We used a step-three procedure from Latent GOLD to analyze the relationship between status membership, general cognitive abilities, and problem solving. We examined how status membership at Time 2 and general cognitive abilities predicted accuracy and speed of problem solving for easy and hard problems. Both easy and hard problem solving abilities (rather than mean problem solving) were examined because statuses differed in their response to difficulty. We used a three-step proportional BCH method. Time 2 status membership was entered as the independent variable. General cognitive abilities (VSWM, inhibition, and basic RT) were entered as covariates. We conducted four separate analyses: two with problem solving accuracy (easy and hard), and two with problem solving speed (easy and hard), as the outcome variable. Problem solving accuracy was treated as a continuous distribution; RTs were treated as a gamma distribution. The procedure is effectively an ANCOVA for each of the accuracy analyses, and a logit regression for each of the speed regressions^1^.

**FIGURE 6:**
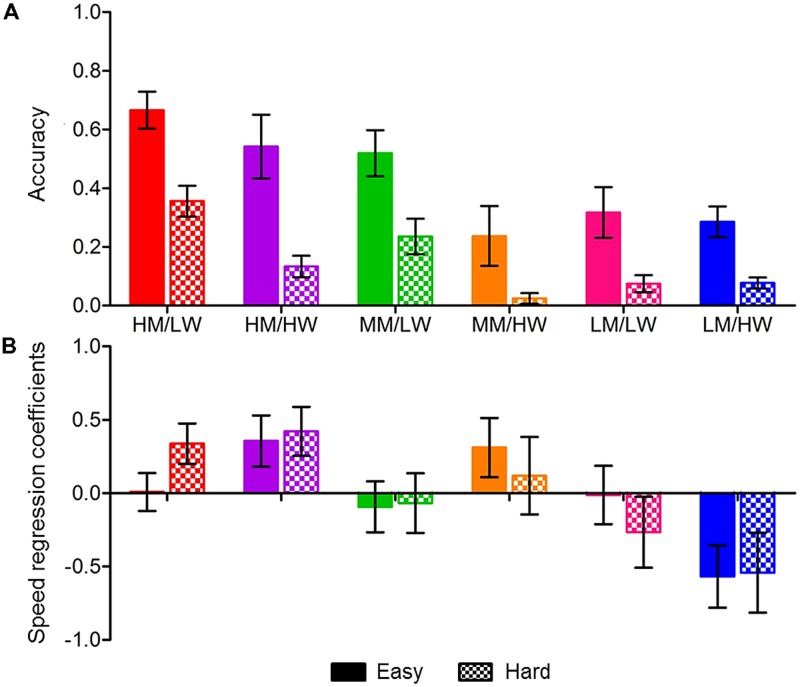
**Three-step analysis outcomes for Time 2 status predicting problem solving (A) accuracy and (B) speed for easy and hard problems**. For **(A)**, higher bars represent higher accuracy. Speed regression coefficients for easy and hard problems are shown in **(B)**; bars above the *x*-axis indicate slower responses and bars below the *x*-axis indicate faster responses. Error bars represent standard error. Statuses are HM/LW (high WM/low worry), HM/HW (high WM/high worry), MM/LW (moderate WM/low worry), MM/HW (moderate WM/high worry), LM/LW (low WM/low worry), and LM/HW (low WM/high worry).

Outcomes of the analyses are reported in **Table 4** and **Figure 6**. **Table 4** reports the estimates for the effects of Status membership, VSWM, inhibition, and basic RT on easy/hard problem solving accuracy and speed. **Table 4** also reports the regression coefficients for VSWM, inhibition, and basic RT. Predicted accuracy and speed regression coefficients for each status are displayed in **Figure 6**. The output in Latent GOLD for gamma distributions presents the coefficients and not the raw RT. For accuracy, we were able to model using normal distributions, for which Latent GOLD presents the accuracy data, rather than regression coefficients. Below, the results of each analysis are presented, and we present a summary of the three-step analyses.

Accuracy for easy problems was significantly predicted by status membership and inhibition, but not VSWM or basic RT (see **Table 4**). The more inhibition errors made, the poorer the students’ problem solving accuracy. **Figure 6** shows that membership to the high WM/low worry, high WM/high worry, and moderate WM/low worry statuses predicted higher accuracy, membership to the moderate WM/high worry, low WM/low worry, and low WM/high worry statuses predicted lower accuracy.

**Table 4:** Multinomial logit regressions of Time 2 status, VSWM, inhibition and RT on problem solving accuracy and speed for easy and hard problems.

	Accuracy						Speed				
	*B*	*SE*	Wald	*df*	*p*	*B*	*SE*	Wald	*df*	*p*
**Easy**									
T2 status			**28.727**	**5**	**<0.001**			**11.383**	**5**	**0.044**
VSWM	0.041	0.027	2.348	1	0.130	-0.023	0.075	0.095	1	0.760
Inhibition	**0.460**	**0.119**	**15.034**	**1**	**<0.001**	-0.198	0.632	0.099	1	0.750
RT	-0.0003	0.0004	0.523	1	0.470	0.001	0.001	0.730	1	0.390
**Hard**									
T2 status			**48.041**	**5**	**<0.001**			**11.825**	**5**	**0.037**
VSWM	**0.042**	**0.018**	**5.675**	**1**	**0.017**	-0.068	0.091	0.562	1	0.450
Inhibition	0.059	0.065	0.821	1	0.370	-0.064	0.610	0.011	1	0.920
RT	0.000	0.0002	0.002	1	0.970	0.001	0.001	0.867	1	0.350

*T2: Time 2. RT: Response Time. Bold indicates p < 0.05.*

*Coefficients (B) and standard errors (SEs) for general cognitive measures, and multivariate Wald tests for all predictors.*

Accuracy for hard problems was significantly predicted by WM-worry status membership, and VSWM. High accuracy was predicted by membership to the high WM/low worry; moderate accuracy was predicted by high WM/high worry status membership; low accuracy was predicted by membership to the moderate WM/high worry, low WM/low worry, and low WM/high worry statuses. For a one-unit increase in VSWM, students were more likely to correctly solve more problems.

Speed of easy problem solving was significantly predicted by WM-worry status membership but not by general cognitive factors. Slow problem solving was predicted by high WM/high worry and moderate WM/high worry status membership. Very fast problem solving was predicted by low WM/high worry status membership. Membership to a low worry status did not appear to predict problem solving speed for easy problems.

Speed of solving for hard problems was predicted by WM-worry status membership. Slow problem solving was predicted by membership to the high WM/low worry and high WM/high worry statuses. Moderate problem solving speed was predicted by membership to the moderate WM/low worry and moderate WM/high worry statuses. Fast problem solving was predicted membership to the low WM/low worry status. Very fast problem solving was predicted by membership to the low WM/high worry status. General cognitive factors did not predict hard problem solving speed.

Overall, the findings show that students in the high WM/low worry status showed the highest accuracy, for both easy and hard problem solving, and average speed for easy, but slower responses for hard problems. The high WM/high worry status displayed slow responses for all problems and high accuracy for easy problems, but average accuracy for hard problems. The moderate WM/low worry status showed high accuracy and moderate speed for easy and hard problems. The moderate WM/high worry status showed inaccurate and slow responses for easy problems and very low accuracy with average RT for hard problems. The low WM/low worry status showed average accuracy and RT for easy problems and low accuracy with fast responses for hard problems. The low WM/high worry status had very fast RT for easy and hard problems, and inaccurate problems solving for hard problems. The findings suggest that for individuals with high worry, problem solving RT is likely to vary depending on WM.

## DISCUSSION

The current study investigated patterns of WM and worry relationships over time, and how these relationships predict problem solving abilities. We were interested in whether WM and/or worry changed over time, and characterizing these patterns of stability/change using LTA. The results are evidence that WM and worry can change over a short space of time, and that initial WM and worry predict patterns of change. The findings demonstrate that WM-worry relationships predict speed and accuracy of algebraic problem solving.

Six distinct patterns of WM-Worry relationships were identified: 3 WM (high, medium, low) × 2 worry (low, high) subgroups. In contrast, Trezise and Reeve (2014) identified four WM-worry relationships at a single time point, which most closely resemble the high WM/low worry, high WM/high worry, moderate WM/low worry, and moderate WM/high worry statuses identified in the present study. Most individuals belonged to the high or moderate WM statuses at Time 1, and membership to the low WM/low worry and low WM/high worry statuses increased at Time 2, suggesting that very low WM emerges over time. Below we first examine changes in WM-worry relationships over time. To explore the effects of both WM and worry on WM-worry relationships, statuses with similar WM or worry levels are compared. Given few individuals are in the low WM statuses at Time 1, we concentrate on high and moderate WM statuses. Second, we examine how WM-worry relationships predict algebraic problem solving abilities by examining the relationships between Time 2 status membership and problem solving characteristics.

### WM-WORRY RELATIONSHIPS

Many students exhibited a stable WM-worry relationship between Time 1 and Time 2. For students whose WM-worry relationship changed over time, the general pattern of change was to move to a status with lower WM, indicating WM capacity declined over time for one-third of students. As expected, the highest stability was seen in high WM/low worry status. Less than half of individuals in the high WM/high worry, moderate WM/low worry, moderate WM/high worry, and low WM/low worry statuses stayed in the same WM-worry status across time, which suggests that, as predicted, both high worry and lower WM were associated with changes in the WM-worry relationship over a short period of time.

To explore the effect of worry to WM-worry changes over time, we compare the Time 1 high WM/low worry and high WM/high worry statuses, as both had a large number at Time 2, and similar WM but different worry levels. For students who initially displayed high WM capacity, those with high worry were more likely to change to a lower WM status over time, suggesting worry reduces WM capacity. These findings support the ACT of anxiety and cognition (Eysenck et al., 2007; Eysenck and Derakshan, 2011), which proposes high worry will reduce WM, as worry consumes processing capacity. The effect of worry on WM also supports Pnevmatikos and Trikkaliotis’ (2013) findings that emotional experience can change cognitive abilities over a short period of time, and more specifically, that negative emotion leads to a decline in cognitive abilities. Moreover, we found that not all students with high worry at Time 1 changed to a lower WM status at Time 2, which suggests other factors may interact with WM and worry to predict changes in WM and worry.

The effects of WM can be explored through comparison of the Time 1 high WM/low worry and moderate WM/low worry statuses, which had similar worry levels but different WM levels^2^. Emotion regulation research suggests that individuals with large WM capacity are better able to regulate their negative emotion and thoughts (Schmeichel et al., 2008; Schmeichel and Demaree, 2010; Hofmann et al., 2012). We therefore predicted that the high WM status would remain stable, and that moderate WM would be associated with increases in worry over time. The high WM status was associated with stability in both WM and worry, and students in the moderate WM status were more likely to change their WM-worry relationship over time. Contrary to our predictions, students in the moderate WM/low worry status were likely to change to the low WM/low worry *or* low WM/high worry statuses, indicating that moderate WM was associated with declines in WM, and *possible* increases in worry. While the findings show some support to the notion that individual with high WM are better at regulating emotion (see Hofmann et al., 2012), this was not true for all students. It is possible that algebraic equations may not elicit negative emotions in some students, as suggested by Young et al. (2012); rather, performing cognitively demanding tasks may have depleted resources, reducing WM capacity. It is also possible that some used cognitive resources to maintain/reduce worry levels (i.e., emotion regulation), which may have depleted cognitive resources and lead to a decline in WM capacity over time. Indeed, Scheibe and Blanchard-Fields (2009) found that after reducing their negative emotions (down-regulation), young adults’ WM capacity decreased. Further research is required to characterize patterns of emotion control and possible short-term effects on cognitive resources.

There were two overall patterns over time: (1) group membership predicted stability/change, and (2) few students moved “up” in group membership, i.e., to a higher WM or low worry group. The study has demonstrated the interactive nature of WM-worry relationships. The findings show support for both anxiety-performance theories (such as the ACT; Eysenck et al., 2007; Eysenck and Derakshan, 2011) and some support for emotion regulation theory (e.g., Hofmann et al., 2012). Together, the patterns of change and consistency with theories, suggest that changes in WM-worry groups represent true changes rather than measurement unreliability or random fluctuation in students’ cognition and/or emotion. Moreover, the interactive nature of these relationships are revealed to be more complicated than can be accounted for by a one-directional perspective (i.e., anxiety affecting cognition, or cognition moderating emotion). The findings emphasize the need for a better description of cognition-emotion interactions that integrates current perspectives.

### PROBLEM SOLVING AND WM-WORRY

Working memory-worry status membership predicted problem solving accuracy and speed. Moreover, the effect of status on problem solving differed as a function of problem solving difficulty. Overall, individuals in higher WM statuses showed more accurate problem solving. Comparison between the three low worry statuses reveals that problem solving accuracy was highest for the high WM/low worry status, and lowest for the low WM/low worry status. These findings indicate algebraic problem solving requires WM, which supports math cognition research that has demonstrated the influence of WM in math problem solving (see Raghubar et al., 2010).

Anxiety-performance theories predict that high anxiety/worry will increase RTs, but only reduce accuracy when task demands are high (e.g., ACT; see Eysenck et al., 2007; Eysenck and Derakshan, 2011). The ACT also predicts a 2-stage process between effort and anxiety: for undemanding task conditions, high anxious individuals are unlikely to use effortful processing, but under demanding task conditions, high anxious individuals engage in effortful processing to improve their task performance (Eysenck and Derakshan, 2011). Our findings suggest a more complex relationship between worry, WM, and problem solving performance. We found that worry and WM interact to predict problem solving. High WM (with low or high worry) was associated with accurate problem solving for easy problems, but for hard problems only high WM with low worry predicted high accuracy. In other words, the cost of difficulty was greater for individuals with high worry. Similarly, high WM with high worry predicted slower problem solving. The differences between the high WM/low worry and high WM/high worry statuses support the ACT in that worry affected speed before accuracy, and the negative effects of anxiety increased with greater task demands.

Examination of the moderate and low WM statuses suggests a pattern that differs to the 2-stage process predicted by the ACT. The moderate WM/high worry status showed slower, less accurate problem solving than the moderate WM/low worry status for easy problems. For hard problem, although the moderate WM/low worry status was more accurate, there was no difference in problem solving speed between the two moderate WM statuses. For individuals with low WM, problem solving was inaccurate, regardless of worry level. However, high worry predicted *very fast* RTs. Ashcraft and Faust (1994) similarly found that a very high math anxious group showed fast, but inaccurate problem solving for demanding arithmetic problems. Our findings suggest that under conditions of high worry, limited cognitive capacity, and high task demands, individuals do not engage in effortful processing; rather, they engage in avoidance and further impair their task performance.

The low WM statuses emerged over time from students initially characterized by high WM/high worry or moderate WM/low worry. Problem solving in the low WM statuses differed to the high WM/high worry and moderate WM/low worry statuses, which suggests that changes in cognition and/or emotion can change problem solving abilities over a short period of time. Therefore, the findings suggest that students with lower WM and/or higher worry are more prone to declines in problem solving abilities over a school day.

### LIMITATIONS AND FUTURE RESEARCH

We examined the cognition-emotion relationship using algebraic stimuli in worry and WM tasks. The context remained constant across the study: the stimulus (algebra) was consistent across both cognition and emotion tasks. It is possible that patterns of change/stability in the cognition-emotion relationship may differ if context altered, for example, if several types of stimuli were presented, or students were instructed to neutralize their emotions.

The moderate WM/high worry status is quite a small status at both time points. For the students initially in the moderate WM/high worry status who changed status membership over time, they moved with respect to WM level, but remained in a high worry status. This suggests that the individuals remain worried across time, although the worry level was less extreme. It is likely that, despite the small number, the subgroup is meaningful. Trezise and Reeve (2014) identified a subgroup with similar WM and Worry, and algebraic problem solving performance characteristics. The present findings support Trezise and Reeve (2014): this small subgroup may reflect the relative prevalence of very high worry with respect to algebra in the general population.

Although WM-worry relationships predicted change in WM/worry, the findings suggest that other factors interact with WM and worry to predict changes. Motivation and attention have been found to predict individual variation in WM and negative affect (Brose et al., 2012), as well as academic achievement (Dweck, 1986; Steinmayr and Spinath, 2009). Further research is needed to better understand the possible influences of cognition and emotion and factors such as motivation and attention, on changes to the WM-worry relationship.

We assessed problem solving abilities at the end of the day. Our findings revealed large variation in both problem solving accuracy RTs and accuracy between individuals. Given that WM and worry differences between individuals increase over time, variations in problem solving accuracy and RTs may also increase over time. The problem solving subgroups may be characterized differently if problem solving was assessed at the beginning of the study, rather than the end. Future research should explore how problem solving abilities change across time, their relation with changing WM-worry relationships, and possible implications for learning ability.

## CONCLUSION

Our findings suggest that the cognition-emotion relationship can change over a short period of time, and that levels of cognition and emotion predict change/stability. Large WM capacity with low worry was associated with stability. Conversely, students with lower WM and/or higher worry are more likely to show declines in WM over time, and possible further increases in worry, indicating their disadvantage is likely to increase over a short period of time. Examination of WM-worry statuses revealed interactions between cognition, emotion, and problem solving abilities. Our findings highlight the impact of changes to the cognition-emotion relationship on problem solving abilities. Specifically, we demonstrate that cognition-emotion relationships show that students’ problem solving abilities are fluid over a short time period, even when context (e.g., domain) remains constant. Developing a model of the interactive nature of cognition-emotion relationships may help to better understand the implications of both cognition and emotion on problem solving abilities.

## Conflict of Interest Statement

The authors declare that the research was conducted in the absence of any commercial or financial relationships that could be construed as a potential conflict of interest.

## Footnotes

1 The analyses differ between accuracy and speed because they were represented by different distribution types.

2 The MWM/HW subgroup had very high worry and a small number, consequently we did not consider the subgroup suitable to compare with the HWM/HW subgroup in order to draw inferences about the effects of WM on WM-worry relationships.

## References

[B1] Alibali M. W. Knuth E. J. Hattikudur S. McNeil N. M. Stephens A. C. (2007). A longitudinal examination of middle school students’ understanding of the equal sign and equivalent equations. *Think. Learn.* 9 221–247 10.1080/10986060701360902

[B2] Alloway T. P. Passolunghi M. C. (2011). The relationship between working memory, IQ, and mathematical skills in children. *Learn. Individ. Differ.* 21 133–137 10.1016/j.lindif.2010.09.013

[B3] Andersson U. Lyxell B. (2007). Working memory deficit in children with mathematical difficulties: a general or specific deficit? *J. Exp. Child Psychol.* 96 197–228. 10.1016/j.jecp.2006.10.001 17118398 10.1016/j.jecp.2006.10.001

[B4] Ashcraft M. H. Faust M. W. (1994). Mathematics anxiety and mental arithmetic performance: an exploratory investigation. *Cogn. Emot.* 8 97–125 10.1080/02699939408408931

[B5] Ashcraft M. H. Kirk E. P. (2001). The relationships among working memory, math anxiety, and performance. *J. Exp. Psychol. Gen.* 130 224–237 10.1037//0096-3445.130.2.224 11409101

[B6] Bakk Z. Tekle F. B. Vermunt J. K. (2013). Estimating the association between latent class membership and external variables using bias-adjusted three-step approaches. *Sociol. Methodol.* 43 272–311 10.1177/0081175012470644

[B7] Bieri D. Reeve R. A. Champion G. D. Addicoat L. Ziegler J. B. (1990). The Faces Pain Scale for the self-assessment of the severity of pain experienced by children: development, initial validation, and preliminary investigation for ratio scale properties. *Pain* 41 139–150 10.1016/0304-3959(90)90018-9 2367140

[B8] Blair K. S. Smith B. W. Mitchell D. G. V. Morton J. Vythilingam M. Pessoa L. (2007). Modulation of emotion by cognition and cognition by emotion. *Neuroimage* 35 430–440 10.1016/j.neuroimage.2006.11.048 17239620 PMC1862681

[B9] Blaye A. Chevalier N. (2011). The role of goal representation in preschoolers’ flexibility and inhibition. *J. Exp. Child Psychol.* 108 469–483 10.1016/j.jecp.2010.09.006 21122878

[B10] Bolck A. Croon M. Hagenaars J. A. (2004). Estimating latent structure models with categorical variables: one-step versus three-step estimators. *Polit. Anal.* 12 3–27 10.1093/pan/mph001

[B11] Bray B. C. Lanza S. T. Collins L. M. (2010). Modeling relations among discrete developmental processes: a general approach to associative latent transition analysis. *Struct. Equ. Modeling* 17 541–569 10.1080/10705511.2010.510043 21572599 PMC3094019

[B12] Brose A. Schmiedek F. Lövdén M. Lindenberger U. (2012). Daily variability in working memory is coupled with negative affect: the role of attention and motivation. *Emotion* 12 605–617 10.1037/a0024436 21787075

[B13] Bull R. Espy K. A. Wiebe S. A. (2008). Short-term memory, working memory, and executive functioning in preschoolers: longitudinal predictors of mathematical achievement at age 7 years. *Dev. Neuropsychol.* 33 205–228. 10.1080/87565640801982312 18473197 10.1080/87565640801982312 PMC2729141

[B14] Canobi K. H. Reeve R. A. Pattison P. E. (1998). The role of conceptual understanding in children’s addition problem solving. *Dev. Psychol.* 34 882–891 10.1037/0012-1649.34.5.882 9779735

[B15] Canobi K. H. Reeve R. A. Pattison P. E. (2003). Patterns of knowledge in children’s addition. *Dev. Psychol.* 39 521–534 10.1037/0012-1649.39.3.521 12760520

[B16] Caviola S. Mammarella I. C. Cornoldi C. Lucangeli D. (2012). The involvement of working memory in children’s exact and approximate mental addition. *J. Exp. Child Psychol.* 112 141–60 10.1016/j.jecp.2012.02.005 22436893

[B17] Celeux G. Soromenho G. (1996). An entropy criterion for assessing the number of clusters in a mixture model. *J. Classif.* 13 195–212 10.1007/BF01246098

[B18] Clark S. Muthén B. (2009). *Relating Latent Class Analysis Results to Variables not Included in the Analysis.* Available at: https://www.statmodel.com/download/relatinglca.pdf

[B19] Collins L. M. Lanza S. T. (2010). *Latent Class and Latent Transition Analysis: With Applications in the Social, Behavioral, and Health Sciences*. NJ, USA: John Wiley & Sons 10.1002/9780470567333

[B20] Conway A. R. A. Kane M. Bunting M. Hambrick D. Z. Wilhelm O. Engle R. W. (2005). Working memory span tasks: a methodological review and user’s guide. *Psychon. Bull. Rev.* 12 769 10.3758/BF03196772 16523997

[B21] Derakshan N. Eysenck M. W. (2009). Anxiety, processing efficiency, and cognitive performance: new developments from attentional control theory. *Eur. Psychol.* 14 9 10.1027/1016-9040.14.2.168

[B22] Dolan C. V. Jansen B. R. J. van der Maas H. L. J. (2004). Constrained and unconstrained multivariate normal finite mixture modeling of piagetian data. *Multivariate Behav. Res.* 39 69–98 10.1207/s15327906mbr3901_3 26759935

[B23] Dweck C. S. (1986). Motivational processes affecting learning. *Am. Psychol.* 41 1040–1048 10.1037/0003-066x.41.10.1040

[B24] Eysenck M. W. Calvo M. G. (1992). Anxiety and performance: the processing efficiency theory. *Cogn. Emot.* 6 409–434 10.1080/02699939208409696

[B25] Eysenck M. W. Derakshan N. (2011). New perspectives in attentional control theory. *Pers. Individ. Dif.* 50 955–960 10.1016/j.paid.2010.08.019

[B26] Eysenck M. W. Derakshan N. Santos R. Calvo M. G. (2007). Anxiety and cognitive performance: attentional control theory. *Emotion* 7 336–353 10.1037/1528-3542.7.2.336 17516812

[B27] Faust M. W. Ashcraft M. H. Fleck D. E. (1996). Mathematics anxiety effects in simple and complex addition. *Math. Cogn.* 2 25–62 10.1080/135467996387534

[B28] Geary D. C. Hoard M. K. Nugent L. (2012). Independent contributions of the central executive, intelligence, and in-class attentive behavior to developmental change in the strategies used to solve addition problems. *J. Exp. Child Psychol.* 113 49–65 10.1016/j.jecp.2012.03.003 22698947 PMC3392437

[B29] Hofmann W. Friese M. Schmeichel B. J. Baddeley A. D. (2011). “Working memory and self-regulation,” in *Handbook of Self-Regulation*, 2nd Edn, *Research, Theory, and Applications* eds Vohs K. D. Baumeister R. F. (New York, US: Guilford Press) 204–225

[B30] Hofmann W. Schmeichel B. J. Baddeley A. D. (2012). Executive functions and self-regulation. *Trends Cogn. Sci.* 16 174–180 10.1016/j.tics.2012.01.006 22336729

[B31] Humberstone J. Reeve R. A. (2008). Profiles of algebraic competence. *Learn. Instr.* 18 354–367 10.1016/j.learninstruc.2007.07.002

[B32] Imbo I. LeFevre J.-A. (2010). The role of phonological and visual working memory in complex arithmetic for Chinese- and Canadian-educated adults. *Mem. Cognit.* 38 176–185 10.3758/MC.38.2.176 20173190

[B33] Imbo I. Vandierendonck A. (2007). The development of strategy use in elementary school children: working memory and individual differences. *J. Exp. Child Psychol.* 96 284–309 10.1016/j.jecp.2006.09.001 17046017

[B34] Kanske P. Heissler J. Schönfelder S. Bongers A. Wessa M. (2011). How to regulate emotion? Neural networks for reappraisal and distraction. *Cereb. Cortex* 21 1379–1388 10.1093/cercor/bhq216 21041200

[B35] Knuth E. J. Alibali M. W. McNeil N. M. Weinberg A. Stephens A. C. (2005). Middle school students’ understanding of core algebraic concepts: equivalence and variable. *Int. Rev. Math. Equ.* 37 68–76 10.1007/BF0255899.E.J

[B36] Krinzinger H. Wood G. Willmes K. (2012). What accounts for individual and gender differences in the multi-digit number processing of primary school children? *Zeitschrift Für Psychologie* 220 78–89. 10.1027/2151-2604/a000099

[B37] Lanza S. T. Collins L. M. (2007). PROC LCA: a SAS procedure for latent class analysis. *Struct. Equ. Modeling* 14 671–694 10.1080/10705510701575602 19953201 PMC2785099

[B38] Lanza S. T. Patrick M. E. Maggs J. L. (2010). Latent transition analysis: benefits of a latent variable approach to modeling transitions in substance use. *J. Drug Issues* 40 93–120 10.1177/002204261004000106 20672019 PMC2909684

[B39] Marcoulides G. A. Gottfried A. E. Gottfried A. W. Oliver P. H. (2008). A latent transition analysis of academic intrinsic motivation from childhood through adolescence. *Educ. Res. Eval.* 14 411–427 10.1080/13803610802337665

[B40] Mattarella-Micke A. Mateo J. Kozak M. N. Foster K. Beilock S. L. (2011). Choke or thrive? The relation between salivary cortisol and math performance depends on individual differences in working memory and math-anxiety. *Emotion* 11 1000–1005 10.1037/a0023224 21707166

[B41] Meeus W. Van de Schoot R. Klimstra T. Branje S. (2011). Personality types in adolescence: change and stability and links with adjustment and relationships: a five-wave longitudinal study. *Dev. Psychol.* 47 1181–1195 10.1037/a0023816 21639626

[B42] Milner B. (1971). Interhemispheric differences in the localization of psychological processes in man. *Br. Med. Bull.* 27 272–277 4937273 10.1093/oxfordjournals.bmb.a070866

[B43] Ng L. K. (2012). “Mathematics anxiety in secondary school students,” in *Proceedings of the 35th Annual Conference of the Mathematics Education Research Group of Australasia, 2012 on Mathematics education: Expanding horizons* (Singapore: National Institute of Education) 570–577 Available at: http://repository.nie.edu.sg/jspui/bitstream/10497/14387/1/MERGA-2012-570-NgLK_a.pdf

[B44] Nylund K. (2007). *Latent Transition Analysis: Modeling Extensions and an Application to Peer Victimization*. Ph.D. thesis, University of California, LA.

[B45] Owens M. Stevenson J. Hadwin J. A. Norgate R. (2014). When does anxiety help or hinder cognitive test performance? The role of working memory capacity. *Br. J. Psychol.* 105 92–101 10.1111/bjop.12009 24387098

[B46] Pnevmatikos D. Trikkaliotis I. (2013). Intraindividual differences in executive functions during childhood: the role of emotions. *J. Exp. Child Psychol.* 115 245–261 10.1016/j.jecp.2013.01.010 23557712

[B47] Raghubar K. P. Barnes M. A. Hecht S. A. (2010). Working memory and mathematics: a review of developmental, individual difference, and cognitive approaches. *Learn. Individ. Differ.* 20 110–122 10.1016/j.lindif.2009.10.005

[B48] Redick T. S. Broadway J. M. Meier M. E. Kuriakose P. S. Unsworth N. Kane M. J. (2012). Measuring working memory capacity with automated complex span tasks. *Eur. J. Psychol. Assess.* 28 164–171 10.1027/1015-5759/a000123

[B49] Richards J. M. Gross J. J. (2000). Emotion regulation and memory: the cognitive costs of keeping one’s cool. *J. Pers. Soc. Psychol.* 79 410–424 10.1037/0022-3514.79.3.410 10981843

[B50] Scheibe S. Blanchard-Fields F. (2009). Effects of regulating emotions on cognitive performance: what is costly for young adults is not so costly for older adults. *Psychol. Aging* 24 217–223 10.1037/a0013807 19290754 PMC2658623

[B51] Schmeichel B. J. Demaree H. A. (2010). Working memory capacity and spontaneous emotion regulation: high capacity predicts self-enhancement in response to negative feedback. *Emotion* 10 739–744 10.1037/a0019355 21038959

[B52] Schmeichel B. J. Volokhov R. N. Demaree H. A. (2008). Working memory capacity and the self-regulation of emotional expression and experience. *J. Pers. Soc. Psychol.* 95 1526–1540 10.1037/a0013345 19025300

[B53] Schoofs D. Wolf O. T. Smeets T. (2009). Cold pressor stress impairs performance on working memory tasks requiring executive functions in healthy young men. *Behav. Neurosci.* 123 1066–1075. 10.1037/a0016980 19824773 10.1037/a0016980

[B54] Simmons F. R. Willis C. Adams A.-M. (2012). Different components of working memory have different relationships with different mathematical skills. *J. Exp. Child Psychol.* 111 139–155 10.1016/j.jecp.2011.08.011 22018889

[B55] Steinmayr R. Spinath B. (2009). The importance of motivation as a predictor of school achievement. *Learn. Individ. Differ.* 19 80–90 10.1016/j.lindif.2008.05.004

[B56] Suárez-Pellicioni M. Núñez-Peña M. I. Colomé A. (2013). Mathematical anxiety effects on simple arithmetic processing efficiency: an event-related potential study. *Biol. Psychol.* 93 517–526 10.1016/j.biopsycho.2013.09.012 24120643

[B57] Tolar T. T. D. Lederberg A. A. R. Fletcher J. M. (2009). A structural model of algebra achievement: computational fluency and spatial visualisation as mediators of the effect of working memory on algebra achievement. *Educ. Psychol.* 29 239–266 10.1080/01443410802708903

[B58] Trezise K. Reeve R. A. (2014). Working memory, worry, and algebraic ability. *J. Exp. Child Psychol.* 121C 120–136 10.1016/j.jecp.2013.12.001 24487226

[B59] Turner M. L. Engle R. W. (1989). Is working memory capacity task dependent? *J. Mem. Lang.* 28 127–154 10.1016/0749-596X(89)90040-5

[B60] Uusimaki L. Nason R. (2004). “Causes underlying pre-service teachers’ negative beliefs and anxieties about mathematics,” in *Proceedings of the 28th Conference of the International Group for the Psychology of Mathematics Education* Vol. 4 (Norway: Bergen University College) 369–376

[B61] Van Dillen L. F. Heslenfeld D. J. Koole S. L. (2009). Tuning down the emotional brain: an fMRI study of the effects of cognitive load on the processing of affective images. *Neuroimage* 45 1212–1219 10.1016/j.neuroimage.2009.01.016 19349235

[B62] Van Dillen L. F. Koole S. L. (2007). Clearing the mind: a working memory model of distraction from negative mood. *Emotion* 7 715–723 10.1037/1528-3542.7.4.715 18039038

[B63] Van Dillen L. F. Koole S. L. (2009). How automatic is “automatic vigilance”? The role of working memory in attentional interference of negative information. *Cogn. Emot.* 23 1106–1117 10.1080/02699930802338178

[B64] Vermunt J. K. (2010). Latent class modeling with covariates: two improved three-step approaches. *Polit. Anal.* 18 450–469 10.1093/pan/mpq025

[B65] Vermunt J. K. Magidson J. (2013a). *Latent GOLD 5.0 Upgrade Manual.* Belmont, MA: Statistical Innocations Inc

[B66] Vermunt J. K. Magidson J. (2013b). *Technical Guide for Latent GOLD 5. 0: Basic, Advanced, and Syntax 1*. Belmont, MA: Statistical Innocations Inc

[B67] Young C. B. Wu S. S. Menon V. (2012). The neurodevelopmental basis of math anxiety. *Psychol. Sci.* 23 492–501 10.1177/0956797611429134 22434239 PMC3462591

